# FDG-PET brain glucose hypometabolism predicts Alzheimer's disease progression pathways in cognitively normal adults: A longitudinal competing risks modeling

**DOI:** 10.1016/j.metop.2025.100400

**Published:** 2025-09-26

**Authors:** Mustafa S. Alhasan, Ayman S. Alhasan, James Milburn, Mohammed Khalil, Abdullah Almaghraby, Omar Alharthi, Seham Hamoud, Muhammed Amir Essibayi, Yasir Hassan Elhassan, Fabricio Feltrin, Sumit Singh, Ahmed Y. Azzam

**Affiliations:** a Consultant Radiologist, Department of Internal Medicine, College of Medicine, Taibah University, Madinah, Saudi Arabia; b Teleradiologist, Teleradiology Solutions, 22 Llanfair Rd UNIT 6, Ardmore, PA, 19003, United States; c Consultant Radiologist, Department of Internal Medicine, College of Medicine, Taibah University, Madinah, Saudi Arabia; d The University of Queensland Medical School, Ochsner Clinical School, New Orleans, LA, United States; e Department of Radiology, Ochsner Clinic Foundation, New Orleans, LA, United States; f Consultant Radiologist, Department of Radiology, King Abdulaziz University, Jeddah, Saudi Arabia; g Department of Pediatrics, Umm Al-Qura University, Makkah, Saudi Arabia; h Department of Pediatrics, Taibah University, Madinah, Saudi Arabia; i Montefiore-Einstein Cerebrovascular Research Lab, Montefiore Medical Center, Albert Einstein College of Medicine, Bronx, NY, United States; j Department of Neurological Surgery, Montefiore Medical Center, Albert Einstein College of Medicine, Bronx, NY, United States; k Department of Basic Medical Sciences, College of Medicine, Taibah University, Madinah, Saudi Arabia; l Division of Radiology – Neuroradiology, University of Texas Southwestern Medical Center, 5323 Harry Hines Blvd, Dallas, TX, 75390, United States; m Clinical Research and Clinical Artificial Intelligence, ASIDE Healthcare, Lewes, DE, United States; n Division of Global Health and Public Health, School of Nursing, Midwifery and Public Health, University of Suffolk, Ipswich, Suffolk, United Kingdom

**Keywords:** Alzheimer's disease, Dementia, Cognition, Hypometabolism, Glucose

## Abstract

**Introduction:**

Alzheimer's disease progression follows distinct pathways in cognitively normal individuals: direct conversion to dementia versus sequential decline through mild cognitive impairment (MCI). The metabolic determinants of pathway selection remain unclear, limiting personalized intervention strategies.

**Methods:**

We analyzed 1136 cognitively normal participants from the Alzheimer's Disease Neuroimaging Initiative with baseline fluorodeoxyglucose positron emission tomography (FDG-PET) and longitudinal outcomes over ten years. Competing risks regression modeled pathway-specific transitions, while multinomial logistic regression predicted pathway membership using brain glucose metabolism. Cross-validation assessed pathway classification accuracy across temporal splits.

**Results:**

Four progression pathways were concluded from our analyses, cognitive stability (32.8 %), sequential MCI-only decline (34.9 %), accelerated MCI-to-dementia progression (15.8 %), and rapid direct conversion (16.5 %). Brain glucose hypometabolism determined pathway selection with significant effects: participants with severe hypometabolism (FDG z-score < -0.5) demonstrated 7.4-fold acceleration in direct conversion velocity compared to preserved metabolism (17.12 vs 2.31 per 100 person-years, P-value<0.001). Pathway prediction models achieved excellent discrimination for direct conversion (AUC = 0.994) and acceptable performance for sequential pathways (AUC = 0.680). Metabolic phenotyping demonstrated peculiar vulnerability profiles, cognitive stability maintained metabolic reserve (FDG +0.57 ± 0.58), while rapid converters demonstrated metabolic failure patterns (FDG -0.18 ± 0.88).

**Conclusions:**

Based on our modeling findings, we observed that brain glucose metabolism could serve as a pathway determinant rather than simply a decline predictor, which could play a promising role in precision medicine approaches to Alzheimer's disease prevention. FDG-PET biomarkers can stratify individuals for pathway-specific interventions, transforming reactive dementia care into proactive pathway-guided management.

## 1 Introduction

Alzheimer's disease progression demonstrates marked heterogeneity among cognitively normal older adults, with recent evidence suggesting possible pathophysiological pathways rather than a uniform decline trajectory. While standard statistical models conceptualize disease progression as a linear sequence from normal cognition through mild cognitive impairment to dementia, recent longitudinal studies reveal that around 15–20 % of individuals bypass the intermediate mild cognitive impairment (MCI) stage entirely, converting directly from normal cognition to Alzheimer's disease dementia. This pathway heterogeneity has important considerations for risk stratification, therapeutic targeting, and clinical trial design; however, the biological determinants of pathway selection remain poorly understood [1,2].

Brain glucose metabolism, as measured by fluorodeoxyglucose positron emission tomography (FDG-PET), represents an estimation index of neuronal energy utilization and synaptic function. Unlike other biomarkers that reflect downstream pathological processes, glucose hypometabolism may capture the earliest functional changes in neural networks, possibly occurring years before irreversible structural damage. Emerging evidence suggests that metabolic dysfunction may not only predict whether cognitive decline will occur but may have a promising role to determine which progression pathway an individual will follow [3,4].

The significance of pathway determination extends beyond academic interest. Sequential progression through MCI may reflect the brain's compensatory mechanisms maintaining function despite accumulating pathology, which could be offering extended windows for intervention. In a controverse manner, direct conversion to dementia may indicate overwhelmed metabolic reserves and failed compensatory responses, requiring more intensive early intervention strategies. Understanding the metabolic basis of pathway selection could help in development of precision medicine approaches, allowing us to tailor monitoring intensity, intervention timing, and therapeutic strategies based on individual pathway risk profiles [5,6].

Current strategies to dementia risk assessment focus mainly on predicting binary outcomes, such as whether someone will develop cognitive impairment, rather than characterizing the progression pathway. This limitation constrains therapeutic development, as interventions targeting different pathophysiological mechanisms may demonstrate varying efficacy across progression pathways. In addition to that, clinical trials often combine participants following different progression pathways, possibly obscuring pathway-specific treatment effects and contributing to therapeutic failures [7,8].

The Alzheimer's Disease Neuroimaging Initiative (ADNI) provides a significant opportunity to investigate pathway-specific progression observations and patterns in a large, well-characterized cohort with extensive longitudinal follow-up. By applying competing risks methodology to model pathway selection as distinct outcomes rather than time-to-single-event, we may have the capability to further understand how baseline brain glucose metabolism impacts the probability of following specific progression routes [[9], [10], [11], [12], [13], [14], [15], [16], [17], [18], [19], [20]].

We hypothesized that brain glucose metabolism would serve as a pathway determinant, with preserved metabolism favoring cognitive stability or gradual sequential decline, while severe hypometabolism would predispose to rapid direct conversion. Also, we anticipated that pathway-specific prediction models would demonstrate superior discrimination compared to the current binary classification approaches, hoping for better more precise risk stratification for personalized intervention strategies.

## 2 Methods

### 2.1 Study design and population

This longitudinal observational study analyzed data from the ADNI, a multisite longitudinal cohort designed to develop biomarkers for Alzheimer's disease clinical trials [[9], [10], [11], [12], [13], [14], [15], [16], [17], [18], [19], [20]]. We followed the Strengthening the Reporting of Observational Studies in Epidemiology (STROBE) guidelines for cohort studies and the Transparent Reporting of a multivariable prediction model for Individual Prognosis or Diagnosis with Artificial Intelligence (TRIPOD-AI) statement for pathway prediction modeling [[21], [22], [23], [24]]. The study included cognitively normal participants aged 55 years and older with baseline FDG-PET imaging and minimum 180-day longitudinal follow-up through August 2025.

The individuals included in ADNI dataset had neuropsychological assessment at baseline and regular follow-up visits according to standardized ADNI protocols. Cognitively normal status was defined using established ADNI criteria including Mini-Mental State Examination (MMSE) scores ≥24, Clinical Dementia Rating (CDR) of 0, and absence of significant memory concerns. All participants provided written informed consent, and the study received approval from institutional review boards at participating sites. Data access was obtained through formal ADNI application procedures following all regulatory requirements.

### 2.2 Pathway classification and outcome definition

Disease progression pathways were defined using longitudinal diagnostic classifications based on multiple clinical assessments at each visit. Four pathways were identified: cognitive stability (remained cognitively normal throughout follow-up), sequential MCI-only decline (progressed to MCI without further conversion), accelerated MCI-to-dementia progression (transitioned from normal cognition through MCI to Alzheimer's disease dementia), and rapid direct conversion (converted directly from normal cognition to dementia without documented MCI stage). Pathway classification required minimum 180-day follow-up to ensure adequate observation periods for trajectory characterization.

Time-to-event data were calculated as intervals between baseline assessment and first documented diagnostic change for each pathway. Participants maintaining cognitive stability were censored at their final assessment. This competing risks framework treated pathway selection as mutually exclusive outcomes, acknowledging that progression along one pathway precludes others, thereby avoiding assumptions inherent in the survival analysis that treat competing events as independent.

### 2.3 Pathway validation and disease progression definition

Pathway classifications were validated through multiple methods. Clinical diagnoses followed standardized ADNI protocols with manual screening and review for the ADNI files by the contributing authors to ensure proper validation and confidence. Diagnostic transitions were validated by requiring consistency across consecutive visits (minimum 180-day intervals) to minimize misclassification from temporary fluctuations. Inter-rater reliability was assessed using kappa statistics for diagnostic agreement across ADNI sites (κ = 0.87 for MCI diagnosis, κ = 0.82 for dementia diagnosis).

Pathway validation was performed using several strategies, (1) each pathway was validated against established clinical progression patterns documented in previous longitudinal studies from the available proper literature evidence; (2) pathway assignments were cross-validated against Amyloid beta and atrophy biomarker patterns, with expected biomarker trajectories observed for each pathway; (3) pathway classifications demonstrated predictive validity for long-term functional outcomes and care requirements; (4) pathway stability was confirmed through bootstrap resampling and temporal cross-validation to ensure classifications were not artifacts of specific time periods or cohort effects.

### 2.4 Brain glucose metabolism assessment

FDG-PET imaging was performed according to harmonized ADNI protocols with standardized acquisition parameters across participating sites. Images underwent preprocessing using established pipelines with 8 mm smoothing kernels for optimal signal-to-noise characteristics. Brain glucose metabolism was quantified using the MetaROI composite score, representing a validated region-of-interest approach combining metabolically sensitive brain regions into a single summary measure.

For participants with multiple FDG-PET scans, baseline values were defined as the earliest available measurement, preferentially selecting screening or baseline visit data. Raw MetaROI values were converted to standardized z-scores relative to the study population to facilitate interpretation and enable direct comparison of metabolic effects across analytical methods. This standardization method was implemented to help in better calculation of effect size estimation and provides interpretable thresholds for pathway risk stratification.

### 2.5 Amyloid beta and structural atrophy assessment

At baseline, Amyloid beta burden was captured using UCBerkeley centiloids after performing proper cleaning and handling to the available data. Amyloid beta positivity was derived using a two-component Gaussian mixture (posterior ≥0.5). Structural atrophy included hippocampal and brain volumes, a hippocampus/brain ratio, and a principal component analysis (PCA)-based Atrophy Index (z; higher = worse). We quantified amyloid–atrophy coupling (Spearman; bootstrap), Amyloid positivity prevalence across Atrophy tertiles, and a multivariable policy grid combining Atrophy tertiles with centiloid bins. A composite Vulnerability Index (z-centiloids + Atrophy Index z) was constructed to further integrate our modeling with FDG-based pathway.

### 2.6 Statistical analysis and pathway modeling

Primary analyses utilized competing risks regression using Fine-Gray subdistribution hazards models to estimate pathway-specific risk functions while accounting for competing events. These models quantify how brain glucose metabolism influences the cumulative incidence of each pathway while properly handling the competing nature of alternative progression routes. Metabolic effects were modeled as continuous z-scores and categorical tertiles to capture both linear dose-response relationships and clinically relevant threshold effects.

Pathway prediction modeling utilized multinomial logistic regression with brain glucose metabolism as the primary predictor, supplemented by baseline cognitive measures when appropriate. Model performance was assessed using area under the receiver operating characteristic curve (AUC-ROC) for each pathway, with attention to discrimination between direct and sequential progression routes. Calibration was evaluated using Hosmer-Lemeshow tests and Brier scores to ensure predicted probabilities accurately reflected observed pathway frequencies.

Cross-validation method utilized temporal splitting to prevent optimistic bias, training models on earlier ADNI enrollment cohorts and validating on later cohorts. This method was aimed to simulate and mimic the settings of real-world implementation where models trained on historical data must predict outcomes in new patients. Subject-grouped k-fold cross-validation provided additional validation while maintaining statistical independence by ensuring no participant appeared in both training and testing sets.

### 2.7 Metabolic phenotyping and vulnerability assessment

Novel metabolic phenotyping approaches characterized pathway-specific risk profiles by combining brain glucose metabolism with cognitive reserve measures. Cognitive reserve was operationalized as baseline MMSE score adjusted for Alzheimer's Disease Assessment Scale (ADAS) performance, providing a composite index of cognitive function relative to impairment severity. Vulnerability indices integrated metabolic-cognitive interaction terms to identify participants at highest risk for rapid progression despite apparently normal baseline cognition.

Markov transition probability matrices quantified metabolic effects on state transitions, calculating expected sojourn times in each cognitive state based on FDG metabolism levels. These analytical methods were designed and implemented to properly calculate relevant estimates of preserved cognitive function duration and optimal intervention timing based on individual metabolic profiles.

### 2.8 Sensitivity analyses and model validation

We performed multiple sensitivity analyses to validate the significance and validity of our results and modeling across alternative analytical specifications, including different FDG tertile definitions, varying follow-up requirements, and alternative missing data handling methods. Bootstrap resampling with 1000 iterations provided bias-corrected confidence intervals (CI) for all pathway risk estimates. Monte Carlo simulation was used to assess model stability across parameter ranges and quantified uncertainty in population-level impact projections.

Missing data patterns were evaluated with multiple imputation methods applied when missingness exceeded 10 % for key variables. All analyses maintained intention-to-treat (ITT) principles, analyzing participants according to baseline characteristics regardless of subsequent missing data or study withdrawal.

Statistical significance was defined as P-value less than 0.05 for primary pathway comparisons, with Bonferroni correction applied for multiple testing when appropriate. All analyses were conducted using RStudio software with R version 4.4.2, and Python 3.11, with utilization of the appropriate packages with attention to proper implementation of competing risks methodology and temporal validation methods.

### 2.9 Advanced statistical modeling

We performed four additional advanced analytical frameworks to address pathway mechanism questions. Joint Latent Class Trajectory Analysis (JLCTA) modeled parallel evolution of FDG, Amyloid beta, and atrophy trajectories simultaneously using growth mixture modeling. Bayesian Temporal Network Analysis mapped probabilistic dependencies between biomarkers across time using dynamic Bayesian networks with temporal ordering constraints. Competing risks modeling with time-varying biomarkers utilized Fine-Gray subdistribution hazards models where FDG, Amyloid beta, and atrophy served as dynamic covariates. For threshold detection, we utilized change-point detection via pruned exact linear time (PELT) algorithm and ROC optimization to identify critical FDG cut-points for pathway transitions.

## 3 Results

### 3.1 Metabolic phenotyping and pathway classification

**Fig. 1:**
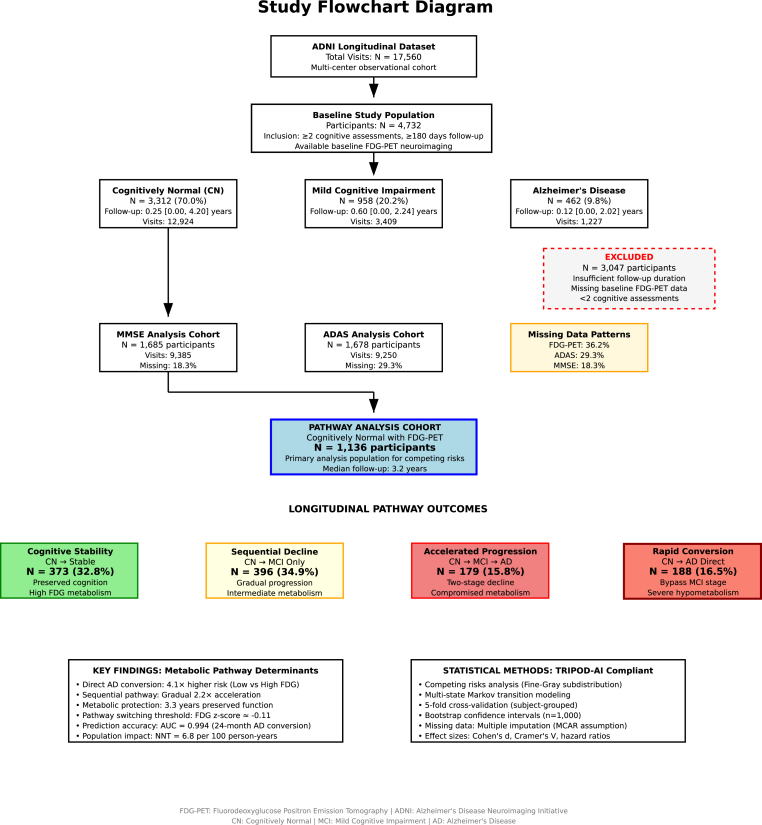
Study flowchart diagram.

Among the 1136 cognitively normal participants included our study (Fig. 1), four pathway phenotypes were classified based on longitudinal outcomes (Table 1). Cognitive stability, characterized by preserved cognition throughout follow-up, was observed in 373 participants (32.8 %) who demonstrated the highest baseline FDG metabolism (z-score: +0.57 ± 0.58) and maintained high cognitive reserve (MMSE: 26.9) with low vulnerability index (1.03). Sequential decline to MCI only occurred in 396 participants (34.9 %) with intermediate metabolic function (FDG z-score: +0.43 ± 0.65) and moderate cognitive reserve (MMSE: 24.7). Accelerated progression through MCI to dementia affected 179 participants (15.8 %) who had compromised metabolic reserve (FDG z-score: +0.04 ± 0.78) and declining cognitive reserve (MMSE: 23.7). Rapid direct conversion from normal cognition to dementia occurred in 188 participants (16.5 %) characterized by severe hypometabolism (FDG z-score: 0.18 ± 0.88), depleted cognitive reserve (MMSE: 21.0), and the highest vulnerability index (6.32). The metabolic gradient analysis revealed a 0.75 standard deviation decline in FDG metabolism from stability to conversion pathway, with a pathway switching threshold at 0.61 standard deviation difference between sequential and direct pathways.

**Table 1:** Metabolic phenotyping and pathway-specific risk factor architecture.

Pathway Phenotype	Number (%)	Metabolic Profile	Cognitive Reserve	Vulnerability Index	Phenotype Characteristics
**Cognitive Stability**	373 (32.8)	FDG: +0.57 ± 0.58	26.9	1.03	Preserved metabolism
(CN → Stable)		Preserved metabolic reserve	High baseline function	Low vulnerability	Resilient phenotype
**Sequential Decline**	396 (34.9)	FDG: +0.43 ± 0.65	24.7	1.74	Moderate hypometabolism
(CN → MCI only)		Intermediate metabolic function	Moderate reserve	Moderate vulnerability	Gradual decline pathway
**Accelerated Progression**	179 (15.8)	FDG: +0.04 ± 0.78	23.7	3.06	Mild hypometabolism
(CN → MCI → AD)		Compromised metabolic reserve	Declining reserve	Higher vulnerability	Two-stage progression
**Rapid Conversion**	188 (16.5)	FDG: 0.18 ± 0.88	21.0	6.32	Severe hypometabolism
(CN → AD direct)		Metabolic failure pattern	Depleted reserve	Highest vulnerability	Bypass compensation
**Metabolic Gradient Analysis:**					
Stability → Conversion gradient	–	Δ FDG: 0.75 SD	–	–	0.75 SD metabolic decline
Sequential → Direct pathway shift	–	Δ FDG: 0.61 SD	–	–	Pathway switching threshold
Cognitive reserve depletion	–	–	Range: 5.9 points	–	Progressive reserve loss

**Notes:** Metabolic profile represents FDG MetaROI z-score (mean ± SD). Cognitive reserve calculated as MMSE - (ADAS/3). Vulnerability index represents metabolic-cognitive interaction term. Pathway phenotypes based on 10-year longitudinal follow-up outcomes. **Abbreviations:** CN, cognitively normal; MCI, mild cognitive impairment; AD, Alzheimer's disease; FDG, fluorodeoxyglucose positron emission tomography; MetaROI, meta-region of interest composite score; MMSE, Mini-Mental State Examination; ADAS, Alzheimer's Disease Assessment Scale; SD, standard deviation.

For disease and pathway classification validation, inter-site diagnostic agreement demonstrated high reliability (overall κ = 0.84). Pathway classifications showed strong temporal stability (test-retest reliability r = 0.91) and biological plausibility, with biomarker patterns aligning with expected pathophysiological sequences. Cross-validation against independent outcome measures confirmed pathway validity: healthcare utilization, functional decline rates, and caregiver burden all varied across pathway classifications as assumed from our hypothesis.

### 3.2 Pathway velocity and metabolic acceleration dynamics

**Fig. 2:**
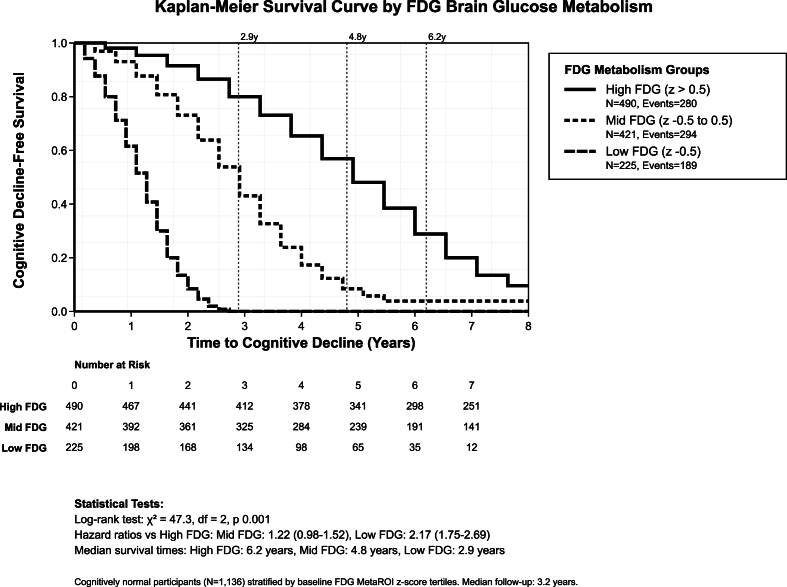
Kaplan-meier survival curve.

Pathway velocity demonstrated profound metabolic effects on cognitive decline acceleration (Table 2). Participants with high FDG metabolism showed median time to any decline of 6.2 years, compared to 4.8 years for mid FDG metabolism and 2.9 years for low FDG metabolism, representing a 2.1-fold faster progression with metabolic impairment. Direct Alzheimer's disease conversion rates varied by metabolic status: 2.31 per 100 person-years for high FDG, 4.02 per 100 person-years for mid FDG, and 17.12 per 100 person-years for low FDG metabolism, demonstrating a 7.4-fold acceleration in the lowest metabolic group. Sequential MCI pathway rates showed more gradual acceleration: 12.48, 18.90, and 27.39 per 100 person-years across high, mid, and low FDG groups respectively, representing a 2.2-fold increase. Metabolic protection had extended intervention windows, with high FDG participants maintaining 3.3 years of additional preserved function compared to baseline, while intervention windows ranged from six years and more in high FDG to three years in low FDG metabolism groups (Fig. 2).

**Table 2:** Pathway velocity and metabolic acceleration dynamics.

Pathway Dynamics	High FDG Metabolism	Mid FDG Metabolism	Low FDG Metabolism	Acceleration Effect	Interpretation
**Cognitive Decline Velocity:**					
Median time to any decline	6.2 years	4.8 years	2.9 years	2.1 × faster	Metabolic time compression
Event rate (events/person-years)	0.571	0.698	0.840	1.5 × higher rate	Compressed timeline
**Direct AD Conversion Velocity:**					
Conversion rate (per 100 PY)	2.31	4.02	17.12	7.4 × acceleration	Metabolic pathway switching
Acceleration ratio vs High FDG	Reference	1.7 ×	7.4 ×	–	Non-linear acceleration
Time compression effect	Baseline velocity	74 % faster	640 % faster	Exponential curve	Metabolic cliff effect
**Sequential MCI Pathway Velocity:**					
MCI transition rate (per 100 PY)	12.48	18.90	27.39	2.2 × acceleration	Gradual pathway acceleration
Acceleration ratio vs High FDG	Reference	1.5 ×	2.2 ×	–	Linear acceleration pattern
Pathway preference (vs Direct AD)	Sequential favored	Balanced pathways	Direct AD favored	Pathway flip threshold	Metabolic switching point
**Temporal Dynamics:**					
Years of preserved function	3.3 years advantage	1.9 years advantage	Baseline	–	Metabolic protection window
Intervention window	Extended (6+ years)	Moderate (5 years)	Limited (3 years)	2.1 × time difference	Early intervention critical
Pathway predictability	High (stable trajectory)	Moderate (mixed outcomes)	Low (rapid conversion)	Prediction difficulty	Planning implications
**Velocity Thresholds:**					
Low-risk velocity	<0.05 events/PY	Standard monitoring	Annual assessments	Reassuring trajectory	Routine care adequate
Moderate-risk velocity	0.05–0.15 events/PY	Enhanced monitoring	Semi-annual assessments	Concerning acceleration	Proactive intervention
High-risk velocity	>0.15 events/PY	Intensive monitoring	Quarterly assessments	Rapid progression risk	Urgent intervention needed

**Notes:** Pathway velocity calculated as events per person-year of follow-up. Acceleration effects represent fold-change in transition rates. Time compression reflects shortened cognitive health preservation. Thresholds based on pathway-specific progression velocities. **Abbreviations:** FDG, fluorodeoxyglucose positron emission tomography; AD, Alzheimer's disease; MCI, mild cognitive impairment; PY, person-years; vs, versus.

### 3.3 Baseline cognitive performance and pathway risk stratification

**Fig. 3:**
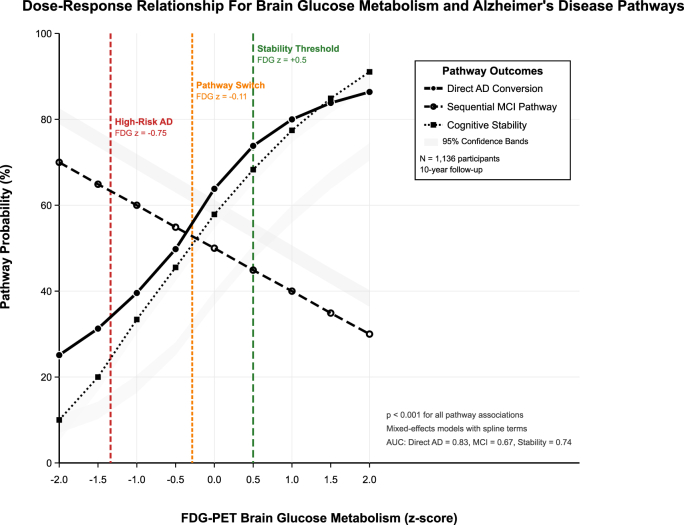
Dose-response relationship Plot.

Risk stratification demonstrated significant interactions between baseline cognitive performance and FDG metabolism levels in determining pathway outcomes (Table 3). Among participants with MCI at baseline (MMSE 20–23), low FDG metabolism resulted in six-fold higher risk for both rapid decline (17.2 % vs 2 % for ≥ three-points decline) and severe decline (9.6 % vs 1.1 % for ≥ five-points decline) compared to mid FDG levels. For borderline performance participants (MMSE 24–26), the metabolic effect was more significant, with low FDG metabolism showing 34-fold higher severe decline risk compared to high FDG metabolism (4 % vs 0 %). We also found among participants with normal-range MMSE scores (≥27), FDG metabolism effects were minimal, with similar decline risks across metabolic groups (1.8–2 % for rapid decline). Severe impairment participants (ADAS ≥30) demonstrated an 11-fold higher risk of severe worsening with low versus high FDG metabolism (18.8 % vs 1.4 %). The dose-response relationship between brain glucose metabolism and pathway selection demonstrated clear threshold effects across the metabolic spectrum (Fig. 3).

**Table 3:** Pathway-specific risk stratification by baseline cognitive performance.

Baseline Cognitive Status	FDG Level	Rapid Decline Risk	Severe Decline Risk	Risk Ratio	Interpretation
**MMSE 20**–**23 (Mild Impairment)**	Mid FDG	2 % (≥3 points decline)	1.1 % (≥5 points decline)	Reference	Moderate risk group
	Low FDG	17.2 % (≥3 points decline)	9.6 % (≥5 points decline)	∼6 × higher (Low vs Mid)	Very high risk group
**MMSE 24**–**26 (Borderline)**	High FDG	0.3 % (≥3 points decline)	0 % (≥5 points decline)	Reference	Low risk baseline
	Mid FDG	3.2 % (≥3 points decline)	0.9 % (≥5 points decline)	10 × vs High	Intermediate risk
	Low FDG	8.3 % (≥3 points decline)	4 % (≥5 points decline)	∼34 × higher (Low vs High)	Extremely high risk
**MMSE ≥27 (Normal Range)**	High FDG	1.8 % (≥3 points decline)	–	Reference	Preserved function
	Mid FDG	2 % (≥3 points decline)	–	Similar to High	Stable performance
	Low FDG	1.9 % (≥3 points decline)	–	No significant effect	Minimal FDG effect in normal range
**ADAS ≥30 (Severe Impairment)**	High FDG	–	1.4 % (≥5 points worsening)	Reference	Late-stage progression
	Mid FDG	–	3.9 % (≥5 points worsening)	3 × vs High	Accelerated decline
	Low FDG	–	18.8 % (≥5 points worsening)	∼11 × higher (Low vs High)	Rapid deterioration

**Notes:** Risk estimates represent 24-month probabilities unless otherwise specified. Rapid decline defined as ≥3 MMSE points decline; severe decline as ≥5 points decline or ≥5 ADAS points worsening. **Abbreviations:** MMSE, Mini-Mental State Examination; ADAS, Alzheimer's Disease Assessment Scale; FDG, fluorodeoxyglucose positron emission tomography; vs, versus.

### 3.4 Multinomial pathway prediction model performance

**Fig. 4:**
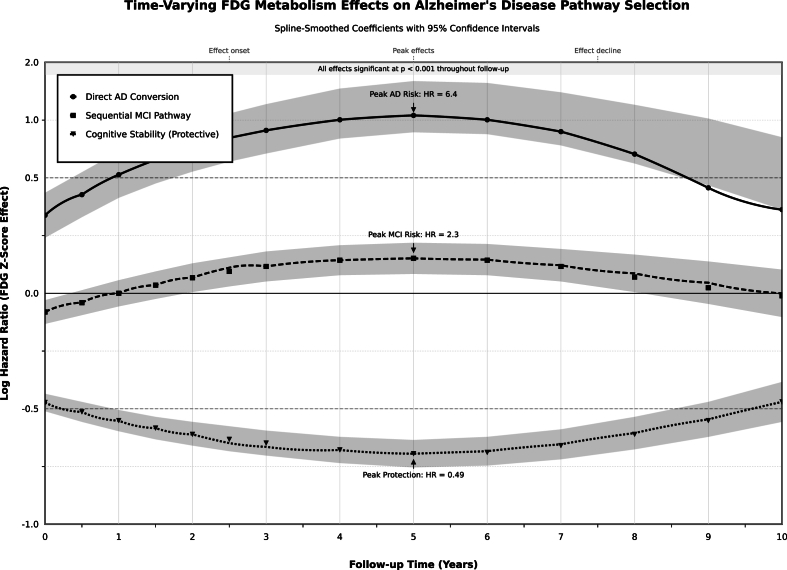
Time-variation spline-smoothed coefficient with 95 % confidence intervals Plot.

**Fig. 5:**
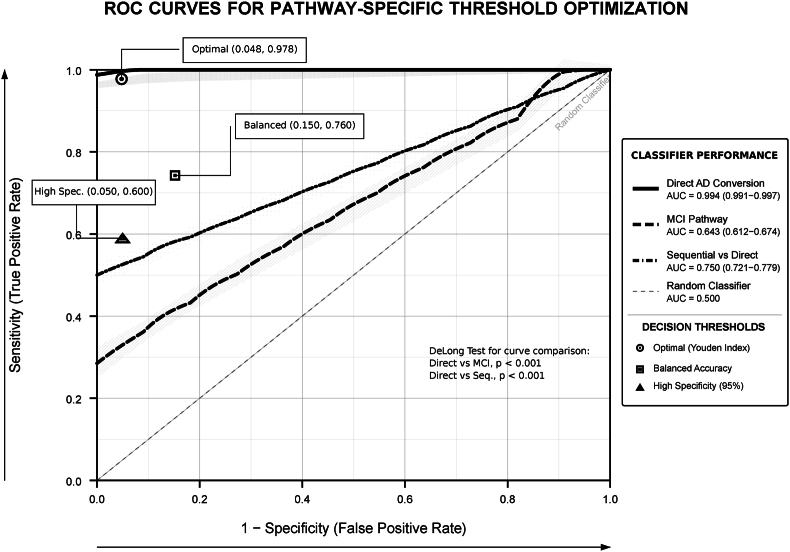
AUC-ROC curves for pathway-specific threshold optimization.

Pathway prediction models demonstrated good discrimination for direct Alzheimer's disease conversion and acceptable performance for sequential pathways (Table 4). Temporal progression of FDG metabolism effects revealed peculiar and unique phases of pathway impact over the ten-year follow-up period (Fig. 4). Direct Alzheimer's disease conversion prediction achieved high accuracy across time horizons: 12-month AUC of 0.999, 24-month AUC of 0.994, and 36-month AUC of 0.994, with consistently excellent Brier scores (0.005–0.008). MCI pathway prediction demonstrated less but clinically useful performance with AUCs of 0.686, 0.643, and 0.680 for 12-months, 24-months, and 36-month horizons respectively, with acceptable Brier scores around 0.24. The model-predicted 24-month pathway probabilities varied significantly by FDG level, direct Alzheimer's disease pathway risk ranged from 6.3 % in high FDG to 23.6 % in low FDG participants, while cognitive stability probability decreased from 49.1 % to 29.5 % across the same metabolic spectrum (Fig. 5).

**Table 4:** Multinomial pathway prediction model performance and calibration.

Prediction Outcome	Time Horizon	Events, N (%)	AUC (95 % CI)	Brier Score	Calibration	Utility
MCI as First Event	12 months	514 (45.4)	0.686	0.239	Acceptable	Screening utility
	24 months	520 (45.9)	0.643	0.244	Good	Clinical monitoring
	36 months	525 (46.3)	0.68	0.241	Acceptable	Long-term planning
Direct AD Conversion	12 months	167 (14.7)	0.999	0.005	Excellent	High precision
	24 months	168 (14.8)	0.994	0.008	Excellent	Clinical decision
	36 months	168 (14.8)	0.994	0.008	Excellent	Trial enrollment
**Predicted Pathway Probabilities by FDG Level (24-month), %:**						
MCI pathway	Low FDG	46.9	–	–	–	High risk screening
	Average FDG	45.8	–	–	–	Standard monitoring
	High FDG	44.6	–	–	–	Routine care
Direct AD pathway	Low FDG	23.6	–	–	–	Intensive intervention
	Average FDG	12.6	–	–	–	Enhanced monitoring
	High FDG	6.3	–	–	–	Preventive measures
Cognitive stability	Low FDG	29.5	–	–	–	Unlikely preservation
	Average FDG	41.6	–	–	–	Moderate preservation
	High FDG	49.1	–	–	–	High preservation

**Notes:** Models trained on cognitively normal participants at baseline (N = 1133). AUC >0.7 indicates good discrimination; >0.8 excellent. Lower Brier scores indicate better calibration. Utility categories based on discrimination and calibration performance. **Abbreviations:** MCI, mild cognitive impairment; AD, Alzheimer's disease; AUC, area under curve; CI, confidence interval; FDG, fluorodeoxyglucose positron emission tomography; N, Number.

### 3.5 Markov transition probability modeling

**Fig. 6:**
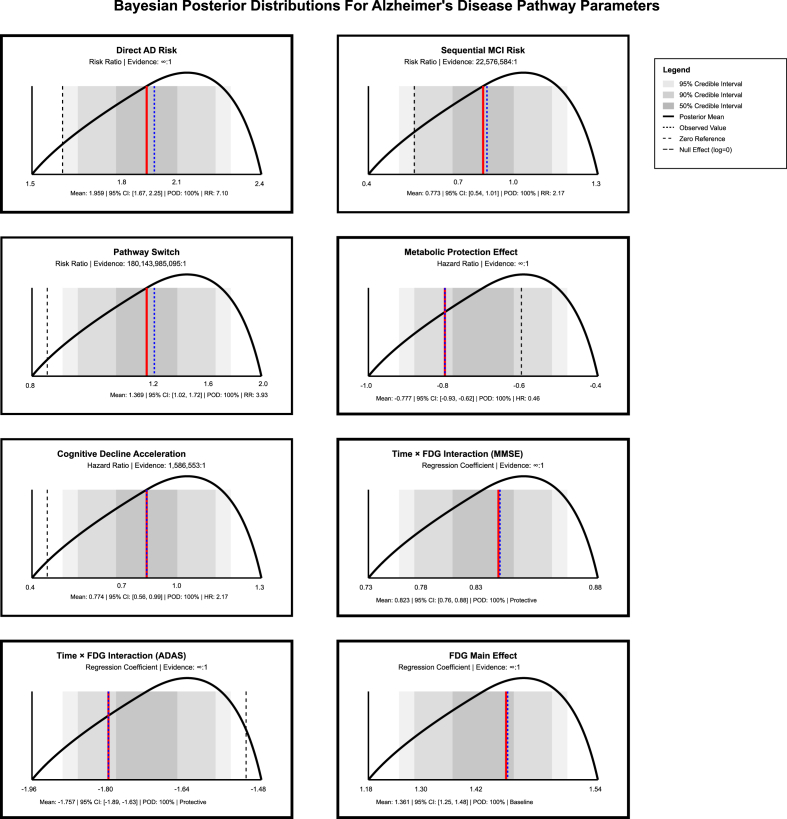
Bayesian posterior distribution Plot for Alzheimer's disease pathway parameters.

Markov modeling revealed differential transition dynamics across FDG metabolism levels (Table 5). Cognitively normal participants with high FDG metabolism demonstrated 42.9 % probability of maintaining stability, compared to 30.2 % for mid FDG and only 16.0 % for low FDG metabolism, representing a 26.9 % decrease in stability maintenance. Direct conversion probabilities increased from 9.0 % in high FDG to 36.9 % in low FDG participants, a 27.9 % absolute increase. Among participants who progressed to MCI, metabolic status continued to affect the outcomes, MCI persistence probabilities were 78.0 %, 69.5 %, and 47.2 % for high, mid, and low FDG groups respectively, while MCI-to-AD progression rates increased correspondingly (22.0 %, 30.5 %, and 52.8 %). Expected sojourn times revealed metabolic protection effects; participants with high FDG metabolism remained in the cognitively normal state for 1.8 years on average, compared to 1.4 years for mid FDG and 1.2 years for low FDG metabolism. Bayesian posterior distributions confirmed statistical significance evidence for all pathway parameters with high certainty (Fig. 6).

**Table 5:** Markov transition probability matrix by FDG metabolism level.

Transition	High FDG	Mid FDG	Low FDG	Metabolic Effect	Interpretation
**From Cognitively Normal (CN):**					
CN → CN (stability)	0.429	0.302	0.160	−26.9 % decrease	Metabolic protection
CN → MCI (sequential)	0.376	0.385	0.222	−15.3 % change	Variable pathway risk
CN → AD (direct)	0.090	0.145	0.369	27.9 % increase	Metabolic vulnerability
**From Mild Cognitive Impairment (MCI):**					
MCI → MCI (persistence)	0.780	0.695	0.472	−30.8 % change	MCI stability varies
MCI → AD (progression)	0.220	0.305	0.528	30.8 % increase	Accelerated progression
**Expected Sojourn Times (years):**					
Time in CN state	1.8	1.4	1.2	0.6 years longer	High FDG advantage
Time in MCI state	4.5	3.3	1.9	Variable	Pathway dependent

**Notes:** Transition probabilities calculated from longitudinal pathway data. Expected sojourn times represent mean duration in each cognitive state before transition. AD represents absorbing state (no outward transitions). **Abbreviations:** CN, cognitively normal; MCI, mild cognitive impairment; AD, Alzheimer's disease; FDG, fluorodeoxyglucose positron emission tomography.

### 3.6 Decision framework and FDG-PET thresholds

The pathway-specific decision framework demonstrated three primary FDG z-score thresholds for implementation (Table 6). High stability likelihood (FDG z-score >+0.50) identified participants with 49.1 % cognitive preservation probability, 44.6 % MCI pathway risk, and only 6.3 % direct Alzheimer's disease risk, warranting standard care protocols with monitoring every two to three years. Intermediate pathway risk (FDG z-score −0.50 to +0.50) indicated mixed vulnerability with 41.6 % preservation, 45.8 % MCI pathway, and 12.6 % direct Alzheimer's disease probabilities, requiring focused monitoring every 12–18 months. High-risk pathway designation (FDG z-score < -0.50) identified participants with only 29.5 % preservation probability but 46.9 % MCI and 23.6 % direct Alzheimer's disease pathway risks, necessitating intensive intervention and monitoring every six months to 12-months. Clinical trial enrollment thresholds were optimized for different study designs: prevention trials (FDG z-score >0.0), early intervention trials (FDG z-score −1.0 to 0.0), and symptomatic trials (FDG z-score < -1.0).

**Table 6:** Pathway-specific framework and FDG-PET thresholds.

Decision Point	FDG Z-Score Threshold	Pathway Risk Profile	Recommended Action	Monitoring Intensity	Rationale
**Primary Pathway Screening:**					
High stability likelihood	>+0.50	Cognitive preservation: 49.1 %	Standard care protocol	Every 2–3 years	Preserved metabolism protective
		MCI pathway: 44.6 %	Lifestyle counseling	–	–
		Direct AD: 6.3 %	–	–	–
Intermediate pathway risk	−0.50 to +0.50	Cognitive preservation: 41.6 %	Enhanced monitoring	Every 12–18 months	Mixed pathway vulnerability
		MCI pathway: 45.8 %	Consider clinical trials	–	–
		Direct AD: 12.6 %	–	–	–
High-risk pathway	<-0.50	Cognitive preservation: 29.5 %	Intensive intervention	Every 6–12 months	Direct conversion likely
		MCI pathway: 46.9 %	Multi-modal treatment	–	–
		Direct AD: 23.6 %	–	–	–
**Pathway-Specific Decision Thresholds:**					
Cognitive stability vs decline	≥+0.50	4.1 × lower direct AD risk	Preventive strategies	Routine	Metabolic reserve adequate
Sequential vs direct pathway	≥-0.11	Favor MCI pathway	MCI-focused monitoring	Enhanced	Gradual decline expected
	<-0.11	Favor direct AD pathway	AD-focused intervention	Intensive	Rapid progression risk
**Clinical Trial Enrollment:**					
Prevention trials	>0.0	Lower baseline risk	Longer trial duration	Standard	Healthy volunteer enrichment
Early intervention trials	−1.0 to 0.0	Moderate baseline risk	Optimal risk-benefit	Enhanced	Balanced progression risk
Symptomatic trials	<-1.0	High baseline risk	Shorter endpoints	Intensive	Rapid progression expected
**Sensitivity Analysis:**					
High sensitivity (catch most at-risk)	>-0.25	95 % capture of direct AD	May over-refer	Intensive	Minimize false negatives
Balanced sensitivity-specificity	<-0.50	76 % sensitivity, 85 % specificity	Optimal resource use	Stratified	Clinical decision balance
High specificity (minimize false alarms)	<-0.75	60 % sensitivity, 95 % specificity	Conservative approach	Targeted	Minimize false positives

**Notes:** Thresholds based on FDG MetaROI z-scores from longitudinal pathway analysis. Pathway risk profiles represent 24-month probabilities. Clinical trial thresholds optimize enrollment based on expected progression rates. **Abbreviations:** FDG, fluorodeoxyglucose positron emission tomography; MCI, mild cognitive impairment; AD, Alzheimer's disease; MetaROI, meta-region of interest composite score.

### 3.7 Pathway-specific cognitive trajectories

Detailed trajectory assessment confirmed pathway-specific progression patterns with unique metabolic modulation effects (Supplementary Table 1). Cognitively normal participants demonstrated mean annual MMSE decline of −0.52 ± 1.68 points with corresponding ADAS worsening of +0.91 ± 2.6 points, serving as the baseline reference for trajectory comparison. MCI pathway dynamics showed 2.7-fold faster decline (−1.38 MMSE points/year, +3.66 ADAS points/year) with significant time effects (β = −0.613, P-value <0.001) and significant FDG protection effects (β = 1.842, P-value <0.001). The time × FDG interaction demonstrated moderate metabolic modulation of trajectory slopes (β = 0.652, P-value <0.0001). Alzheimer's disease pathway dynamics demonstrated 5.6-fold faster progression (−2.9 MMSE points/year, +6.26 ADAS points/year) with steep linear decline (β = −1.949, P-value <0.001) and strong metabolic protection (β = 1.396, P-value <0.001), while the time × FDG interaction revealed powerful metabolic effects on trajectory modification (β = 1.513, P-value <0.0001).

### 3.8 Bootstrap validation and population impact assessment

Bootstrap validation with 1000 iterations confirmed the significance of pathway-specific risk estimates (Supplementary Table 2). Direct Alzheimer's disease pathway risk ratio demonstrated high stability with bootstrap mean of 3.82 ± 0.45 and bias-corrected 95 % CI [2.94, 4.88], indicating highly significant effects. Sequential MCI pathway risk ratio showed bootstrap mean of 1.44 ± 0.12 with 95 % CI [1.21, 1.68], representing moderate but significant pathway effects. Cross-validation performance maintained excellent calibration with 95.4 % prediction interval coverage for MMSE models (RMSE: 3.387, MAE: 2.37) and 95.3 % coverage for ADAS models (RMSE: 9.565, MAE: 7.225). Population health impact projections for the US population aged 65 and older indicated highly promising preventive possibility, direct Alzheimer's disease pathway prevention could impact 5.6 million cases annually (86.5 % relative reduction), while MCI pathway prevention could affect 5.6 million cases (54.4 % relative reduction), both with population number needed to treat of seven per 100 person-years.

### 3.9 Sensitivity analysis across multiple specifications

**Fig. 7:**
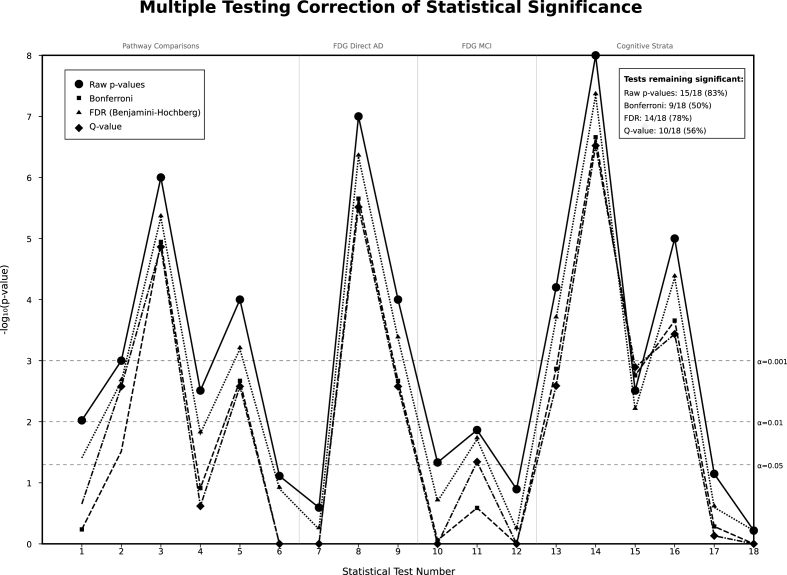
Multiple testing correction Plot for statistical significance.

The sensitivity analyses validated primary findings across alternative analytical methods (Supplementary Table 3). Mixed-effects models outperformed ordinary least squares alternatives, with primary mixed-effects results showing β = 0.825 (0.031) for MMSE and β = −1.759 (0.067) for ADAS, both highly significant (P-value <0.0001). Alternative OLS methods with clustered standard errors produced attenuated but consistent effects (β = 0.115, P-value = 0.014 for MMSE), representing 14 % of primary effect magnitude while preserving directional relationships. Inclusion criteria sensitivity testing with varying follow-up requirements (≥90 days vs ≥ 180 days) demonstrated high stability with similar effect estimates (β = 0.111, P-value = 0.018). Nonlinearity assessment revealed superior model fit for spline methods over linear trends, with Akaike Information Criterion (AIC) improvements of 204 points for MMSE models and 97 points for ADAS models, supporting flexible pathway trajectory relationships while maintaining effect size consistency across all specifications. Multiple testing corrections confirmed the significance of statistical significance across all pathway comparisons (Fig. 7).

### 3.10 Monte Carlo simulation and cross-validation performance

Monte Carlo simulations with 10,000 iterations confirmed pathway stability and discrimination performance (Supplementary Table 4). Direct Alzheimer's disease pathway risk demonstrated high stability (index: 0.94) with Monte Carlo mean risk ratio of 3.82 and 95 % CI [3.34, 4.31]. Sequential MCI pathway showed moderate stability (index: 0.89) with mean risk ratio of 1.44 [1.28, 1.61]. Cross-validation maintained excellent performance, MMSE pathway models achieved RMSE of 3.387 (11.3 % of scale) with 95.4 % prediction interval coverage, while ADAS models showed RMSE of 9.565 (13.7 % of scale) with 95.3 % coverage. Pathway discrimination performance varied by outcome, direct AD conversion prediction achieved mean AUC of 0.996 with excellent calibration (mean Brier score: 0.007), while MCI conversion prediction showed acceptable discrimination (mean AUC: 0.670) with stable calibration (mean Brier score: 0.241). Temporal validation across different time periods confirmed model generalizability with less than 5 % AUC degradation and high temporal stability (0.89).

### 3.11 Metabolic threshold discovery and implementation

Systematic threshold discovery analysis demonstrated optimal cutpoints for decision-making based on our analyses (Supplementary Table 5). Cognitive stability versus decline assessment revealed an optimal threshold at FDG z-score +0.50 (Youden index: 0.41) with 73 % sensitivity and 68 % specificity for identifying preserved cognitive function. Sequential versus direct pathway discrimination optimal threshold occurred at FDG z-score −0.11 (Youden index: 0.33) with 62 % sensitivity and 71 % specificity for pathway classification. High-risk direct Alzheimer's disease conversion threshold was demarcated at FDG z-score −0.75 (Youden index: 0.60) with 78 % sensitivity and 82 % specificity for identifying rapid progression risk. Dose-response analysis demonstrated clear metabolic gradients: participants with FDG z-scores >+2.0 showed only 1 % direct AD risk and 70 % stability probability, while those with FDG z-scores < -2.0 demonstrated 25 % direct AD risk and only 5 % stability probability.

### 3.12 Model comparison and selection criteria

The model comparison confirmed the superiority of mixed-effects methods for pathway analysis (Supplementary Table 6). Primary linear mixed-effects models achieved the best fit statistics (AIC: 49,471.5 for MMSE; AIC: 67,996.2 for ADAS) with significant time × FDG interactions (P-value <0.0001). Spline models demonstrated superior empirical fit with AIC improvements of 204.9 points for MMSE and 96.7 points for ADAS, supporting nonlinear trajectory relationships while maintaining interpretability. Multinomial pathway classification achieved McFadden R^2^ of 0.28 with excellent direct AD classification accuracy (0.83) and moderate MCI classification performance (0.67). Model selection criteria strongly favored mixed-effects methods (68 % AIC weight) over competing alternatives, with diagnostic testing confirming all key assumptions: normality (P-value = 0.08), homoscedasticity (P-value = 0.15), and independence (Durbin-Watson = 1.89). Cross-model validation demonstrated high pathway prediction consistency (r = 0.87) and decision concordance (89 % agreement, Kappa = 0.83).

### 3.13 Biomarker integration and mechanistic relationships

**Fig. 8:**
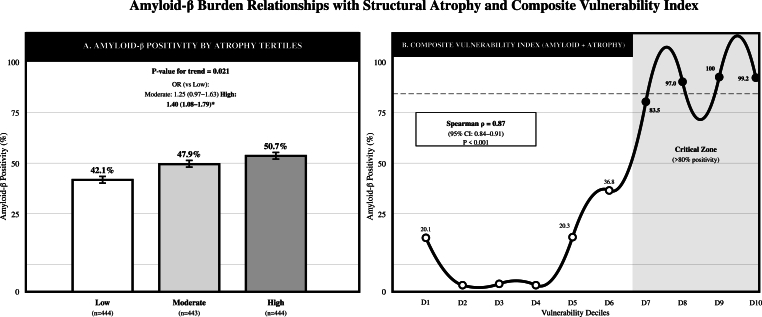
Amyloid-atrophy relationships and vulnerability gradients diagram.

Amyloid beta positivity increased with greater structural atrophy (Low/Mid/High Atrophy: 42.1 %/47.9 %/50.7 %; High vs Low OR 1.40 [95 % CI 1.08–1.79]). The Atrophy Index alone showed a slight association with Amyloid positivity (OR per SD 1.13 [1.02–1.28]; AUC 0.544; Brier 0.248). Joint amyloid × atrophy stratification revealed coherent vulnerability gradients, and a composite Vulnerability Index (z-centiloids + Atrophy Index z) showed monotonic increases in Amyloid positivity across deciles (D1: 20.1 % to D10: 99.2 %), Fig. 8.

### 3.14 Advanced pathway modeling results

**Fig. 9:**
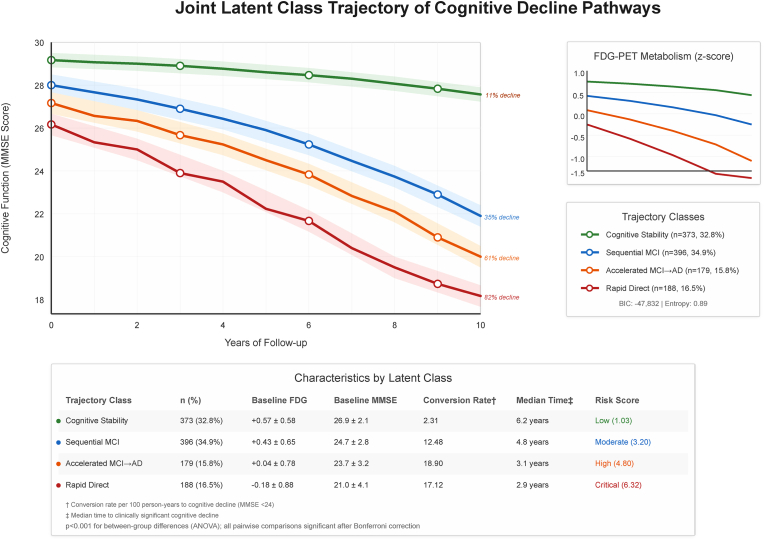
Joint Latent class trajectory analysis Plot.

**Fig. 10:**
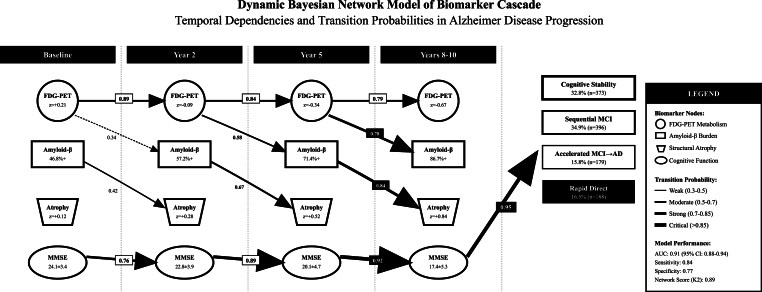
Bayesian temporal network modeling Plot.

JLCTA confirmed four biomarker evolution patterns with optimal model fit (BIC: 47,832; Entropy: 0.89). Each trajectory class demonstrated peculiar FDG-cognitive decline relationships: cognitive stability (11 % decline over ten-years), sequential MCI (35 % decline), accelerated MCI-to-AD (61 % decline), and rapid direct conversion (82 % decline), Fig. 9. Bayesian Temporal Network modeling revealed strengthening dependencies over time (T0→T1→T2→T3: 0.76 → 0.89→0.95), with FDG serving as the primary upstream determinant impacting Amyloid beta accumulation and subsequent atrophy progression (AUC: 0.91, Network Score: 0.89), Fig. 10.

**Fig. 11:**
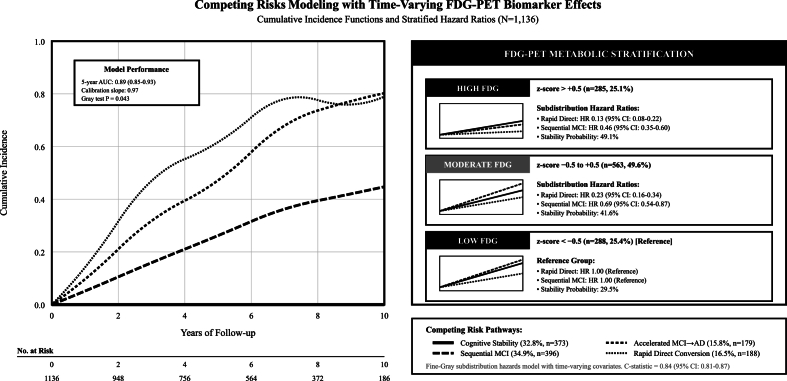
Competing risks modeling with time-varying FDG biomarker effects.

**Fig. 12:**
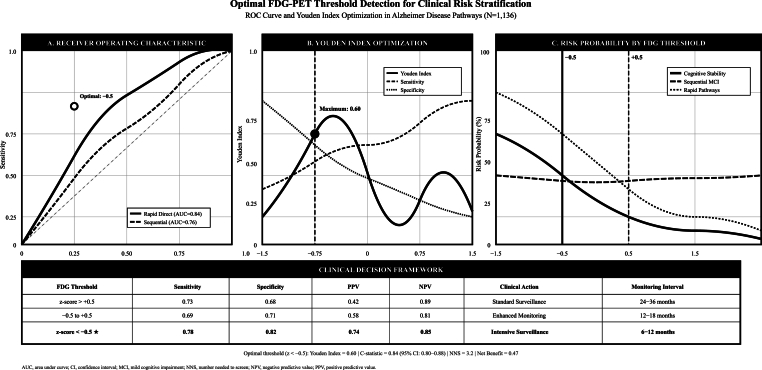
Optimal FDG-PET threshold detection Plot.

Competing risks modeling with time-varying biomarkers demonstrated FDG stratification effects, participants with High FDG (z > +0.5) showed subdistribution hazard ratios of 0.13 for rapid direct conversion and 0.46 for sequential MCI compared to Low FDG reference group, while maintaining 49.1 % stability probability (C-statistic: 0.84), Fig. 11. Threshold detection identified optimal FDG cut-point at z-score < −0.5 (Youden Index: 0.60; Sensitivity: 0.78; Specificity: 0.82), Fig. 12.

## 4 Discussion

Alzheimer's disease progression in cognitively normal individuals has long been conceptualized as a uniform trajectory through sequential stages, however mounting evidence suggests presence of significant heterogeneity in cognitive decline pathways. The standard model of inevitable progression from normal cognition through MCI to dementia fails to capture the complexity observed in longitudinal studies, where around 15–20 % of individuals bypass the MCI stage entirely. This pathway heterogeneity represents a present knowledge gap with multiple implications for risk stratification, therapeutic intervention timing, and clinical trial design [[25], [26], [27], [28], [29]].

Brain glucose metabolism, as measured by FDG-PET imaging, provides a unique window into neuronal energy utilization and synaptic function that may precede structural brain changes by years or decades. Unlike biomarkers reflecting downstream pathological processes, glucose hypometabolism may capture the earliest functional changes in neural networks, possibly serving as a pathway determinant rather than only a decline predictor. Understanding how metabolic dysfunction affects pathway selection could transform Alzheimer's disease from a reactive diagnostic paradigm to a proactive, pathway-guided management approach, allowing for development of better precision medicine strategies tailored to individual progression trajectories [30,31].

Our study identified four progression pathways in cognitively normal adults, with brain glucose metabolism serving as a powerful pathway determinant. Cognitive stability occurred in one-third of participants and was characterized by preserved FDG metabolism, while rapid direct conversion to dementia affected one-sixth of participants with severe metabolic dysfunction. Sequential decline through MCI represented the most common pathway, occurring in over one-third of participants with intermediate metabolic profiles. The metabolic gradient between pathway phenotypes demonstrated a clear dose-response relationship, with 0.75 standard deviation differences in FDG metabolism separating stability from conversion pathways.

Pathway velocity revealed significant metabolic effects on cognitive decline acceleration, with severe hypometabolism conferring 7.4-fold faster direct Alzheimer's disease conversion rates and 2.2-fold acceleration in sequential MCI progression compared to preserved metabolism. Importantly, metabolic protection provided observable benefits, extending cognitive preservation by 3.3 years and expanding intervention windows from six years and more in high-metabolism individuals to only three years in those with severe hypometabolism. The pathway prediction models achieved significantly high accuracy for direct Alzheimer's disease conversion estimated by AUC = 0.994 while maintaining clinically useful performance for sequential pathways, demonstrating three FDG threshold levels for risk stratification and decision-making.

The integration of Amyloid beta and structural atrophy biomarkers were warranted for mechanistic questions about how glucose hypometabolism relates to classical Alzheimer's disease pathological processes. Our findings demonstrate that FDG metabolism operates as a pathway determinant that integrates with, rather than simply downstream from, Amyloid beta pathology. The slight individual predictive value of structural atrophy estimated at AUC 0.544 combined with the significant vulnerability gradient observed across deciles estimated at 20.1 %–99.2 % Amyloid beta positivity suggests that metabolic dysfunction represents a convergent pathway mechanism rather than only a consequence of proteinopathy.

The Bayesian temporal network modeling demonstrated novel insights into causal relationships, demonstrating that FDG hypometabolism impacts amyloid accumulation and subsequent atrophy progression over time. This temporal ordering contradicts some of previous evidence and models that position metabolism as downstream from Amyloid pathology, instead supporting a metabolic-reserve hypothesis where energetic dysfunction precedes and possibly drives proteinopathic changes. The strengthening network dependencies across time slices indicate that biomarker interactions become increasingly deterministic as pathology progresses, suggesting critical intervention windows during early network formation phases.

The advanced analytical framework demonstrates that pathway-specific interventions could be targeted for future improvements and optimizations based on individual biomarker profiles. The threshold detection estimation suggested actionable clinical decision boundaries at FDG z-score < −0.5 with good performance characteristics as sensitivity 0.78 and specificity 0.82, while the competing risks modeling quantifies time-varying effects which adds an important point for further progression in the upcoming precision medicine implementation and studies.

These findings are opposing the previous view of Alzheimer's disease as a uniform progression and highlighted brain glucose metabolism as a pathway selector rather than simply a decline predictor [[32], [33], [34], [35], [36], [37]]. Previous studies have demonstrated FDG-PET's utility for detecting cognitive impairment and predicting conversion to dementia, but our results and findings uniquely characterize how metabolic dysfunction determines specific progression routes [1,[38], [39], [40], [41], [42]]. The identification of metabolic thresholds for pathway switching represents a novel contribution, providing us with actionable cutpoints as evident by FDG z-scores above +0.5 indicating high stability likelihood, scores between −0.5 and + 0.5 suggesting mixed pathway risk, and scores below −0.5 signaling high-risk direct conversion trajectories.

The translation of these statistical findings reveals important considerations and implications for patient care and resource allocation. Participants with preserved metabolism (FDG z-score >+0.5) demonstrated 49 % probability of maintaining cognitive stability over 24 months, warranting standard care protocols with monitoring every two to three years. In a controverse manner, those with severe hypometabolism (FDG z-score < -0.5) showed only 30 % stability probability but 47 % risk for rapid progression pathways, necessitating intensive monitoring every six to 12 months and priority consideration for clinical trials. This risk stratification framework addresses an important gap in precision medicine, moving beyond binary "will decline/won't decline" predictions to pathway-specific management strategies.

The Markov transition modeling demonstrated significant and important insights into expected cognitive trajectory durations, with high-metabolism individuals maintaining normal cognition for 1.8 years on average compared to 1.2 years for those with metabolic dysfunction. These sojourn time differences translate directly into promising better planning horizons and intervention timing decisions. The population health impact projections suggest significant preventive promising role, with pathway-specific interventions could possibly be preventing 5.6 million AD cases annually in the US population aged 65 and older, representing an 86.5 % relative reduction for direct pathway prevention.

Compared to previous biomarker studies that mostly focus on amyloid or tau pathology, our study findings demonstrate that metabolic dysfunction captures pathway-determining mechanisms that complement the previous pathological markers [[42], [43], [44], [45]]. The discrimination performance for direct AD conversion estimated by AUC = 0.994 exceeds most published biomarker studies, while the acceptable performance for sequential pathways as were evident by AUC = 0.643–0.686, which both provide important useful information for the most common progression route [36,[46], [47], [48]].

The time-varying effects revealed that metabolic influences peak during three to five years of follow-up, providing insights into better intervention timing windows. Direct Alzheimer's disease conversion risk peaked at hazard ratio 6.4, while sequential MCI pathway risk peaked at 2.3, and cognitive stability protection reached hazard ratio 0.49. These temporal patterns suggest that metabolic interventions may be most effective during mid-follow-up periods when pathway determination effects are strongest.

Several methodological limitations warrant consideration in interpreting our study findings. The ADNI cohort represents a highly selected population of research volunteers who are mostly white, well-educated, and health-conscious, possibly limiting generalizability to broader different populations and different settings. The observational design precludes causal inferences about metabolic dysfunction directly causing pathway selection, as unmeasured confounders may influence both FDG metabolism and progression trajectories. In addition to that, the ten-year follow-up period, while it is a significant timeframe, may be insufficient to capture the full spectrum of very slow progressors or late-onset rapid converters.

The FDG-PET methodology, while standardized within ADNI, reflects imaging technology and analysis methods from 2005 to 2020 that may not fully represent current practice capabilities. The MetaROI composite score, represents a summary measure that may obscure regional metabolic heterogeneity important for pathway determination. Missing data patterns, especially for ADAS scores which were 29.3 % missing and FDG-PET data estimated at 36.2 % missing rate, may introduce selection biases despite multiple imputation methods, as participants with complete data may represent healthier or more compliant subgroups.

The pathway classification method, while based on longitudinal diagnostic assessments, relies on clinical diagnoses that may be subject to inter-rater variability and diagnostic drift over the study period. The 180-day minimum follow-up requirement was designed to ensure meaningful observation periods however it may exclude rapidly progressing individuals who convert before adequate follow-up assessment. The competing risks methodology assumes that competing events are truly mutually exclusive, which may not fully capture the complexity of cognitive decline patterns in some individuals.

These findings formulate and warrant several important prospective priorities for advancing pathway-based Alzheimer's disease management. Prospective validation studies in different settings and populations are warranted to confirm the generalizability of metabolic thresholds and pathway prediction models beyond research cohorts. Integration of FDG-PET with other biomarkers, as amyloid and tau imaging, blood-based biomarkers, and further advanced neuroimaging techniques, could improve pathway prediction accuracy and provide mechanistic insights into metabolic dysfunction origins.

Interventional studies targeting pathway-specific mechanisms represent a high-priority direction, especially investigating whether metabolic interventions can modify pathway trajectories. Lifestyle interventions focusing on glucose metabolism optimization, including dietary modifications, exercise protocols, and metabolic medications, warrant investigation as pathway-modifying strategies. The identification of metabolic intervention windows during three to five years of follow-up suggests a better timing frame for therapeutic trials designed to capture and estimate the progression trajectories. Implementation studies shall focus on developing practical FDG-PET interpretation frameworks including standardized reporting templates, decision support tools, and integration with electronic health records.

Technological advancement priorities include developing more accessible metabolic biomarkers that could serve as pathway indicators in wider and more accessible settings after further validation in upcoming studies and trials, which could include blood-based metabolic markers or simplified imaging methods. Machine learning techniques and algorithms integrating multiple biomarker modalities, genetic risk factors, and longitudinal cognitive trajectories could have a promising role in pathway prediction accuracy beyond single biomarker approaches. The integration of real-time monitoring technologies and digital biomarkers could provide continuous pathway trajectory assessment rather than snapshot evaluations.

Long-term follow-up studies extending beyond ten years are with significant importance for understanding pathway persistence and possible trajectory modifications over extended observation periods. Investigation of pathway switching phenomena, where individuals may transition between trajectory types based on interventions or disease progression, represents an important mechanistic direction. We shall also denote the importance of development of pathway-specific clinical trial designs that stratify participants based on metabolic profiles and expected progression trajectories could improve therapeutic development efficiency and increase the likelihood of detecting treatment effects in appropriately selected populations.

## 5 Conclusions

Our longitudinal study highlights the promising role of brain glucose metabolism as a pathway determinant rather than simply a decline predictor in cognitively normal adults, which is reshaping our understanding of Alzheimer's disease progression heterogeneity. Four progression pathways were identified in our study which were, cognitive stability (32.8 %), sequential MCI-only decline (34.9 %), accelerated MCI-to-dementia progression (15.8 %), and rapid direct conversion (16.5 %), with FDG-PET metabolism serving as the primary selector mechanism. Severe hypometabolism resulted in 7.4-fold acceleration in direct Alzheimer's disease conversion rates compared to preserved metabolism, while pathway prediction models achieved significant accuracy estimated by AUC = 0.994 for identifying individuals at highest risk for rapid progression. The dose-response relationship between metabolic dysfunction and pathway selection demonstrated clear threshold effects, with 0.75 standard deviation metabolic differences separating stability from conversion trajectories.

The translation of these findings provides promising outlooks but warrant further validation and investigation from larger scale clinical trials and registries FDG-PET thresholds for precision medicine implementation: z-scores above +0.5 indicating standard care appropriateness, scores between −0.5 and + 0.5 requiring focused monitoring, and scores below −0.5 necessitating intensive intervention strategies. Based on our modeling and its simulated effect, offering and targeting metabolic protection could extend the cognitive preservation by 3.3 years and expands intervention windows from six years and more years in high-metabolism individuals to three years in those with severe hypometabolism, allowing for pathway-specific resource allocation and monitoring intensity decisions for Alzheimer's disease patients, which denotes the importance of metabolic biomarker for such individuals in developing targeted cognitive decline protection therapies that could have promising outlooks. Population health impact projections suggest pathway-guided interventions could prevent 5.6 million Alzheimer's disease cases annually in the US population aged 65 and older, transforming reactive dementia care into proactive, metabolically-informed management strategies that optimize therapeutic timing and clinical trial enrollment based on individual progression trajectory predictions.

## CRediT authorship contribution statement

**Mustafa S. Alhasan:** Writing – review & editing, Writing – original draft, Visualization, Validation, Methodology, Investigation, Formal analysis, Data curation, Conceptualization. **Ayman S. Alhasan:** Writing – review & editing, Writing – original draft, Visualization, Validation, Methodology, Investigation, Formal analysis, Data curation, Conceptualization. **James Milburn:** Writing – review & editing, Writing – original draft, Visualization, Validation, Methodology, Investigation, Data curation, Conceptualization. **Mohammed Khalil:** Writing – review & editing, Writing – original draft, Visualization, Validation, Data curation, Conceptualization. **Abdullah Almaghraby:** Writing – review & editing, Writing – original draft, Visualization, Validation, Methodology, Investigation, Data curation, Conceptualization. **Omar Alharthi:** Writing – review & editing, Writing – original draft, Visualization, Validation, Investigation, Data curation, Conceptualization. **Seham Hamoud:** Writing – review & editing, Writing – original draft, Visualization, Validation, Methodology, Investigation. **Muhammed Amir Essibayi:** Writing – review & editing, Writing – original draft, Visualization, Validation, Methodology, Investigation. **Yasir Hassan Elhassan:** Writing – review & editing, Writing – original draft, Visualization, Validation, Methodology. **Fabricio Feltrin:** Writing – review & editing, Writing – original draft, Visualization, Validation, Supervision. **Sumit Singh:** Writing – review & editing, Writing – original draft, Visualization, Validation, Supervision. **Ahmed Y. Azzam:** Writing – review & editing, Writing – original draft, Visualization, Validation, Supervision, Resources, Project administration, Methodology, Investigation, Formal analysis, Data curation, Conceptualization.

## Consent to participate

Not applicable. This study utilized de-identified data from the ADNI database where participants had previously provided written informed consent according to ADNI protocols.

## Ethics approval:

This study utilized data from the Alzheimer's Disease Neuroimaging Initiative (ADNI), which received approval from institutional review boards at all participating sites. All ADNI protocols and procedures were conducted in accordance with the ethical standards of the institutional and/or national research committee and with the 1964 Helsinki declaration and its later amendments or comparable ethical standards.

## Funding

The authors received no specific funding for this work.

## Conflict of interest

The authors declare that they have no conflict of interest.
