# Supplementary material for: FDG-PET brain glucose hypometabolism predicts Alzheimer's disease progression pathways in cognitively normal adults: A longitudinal competing risks modeling
Source: Metabol Open. 2025 Sep 26;28:100400. doi: 10.1016/j.metop.2025.100400 (PMC12516547; doi:10.1016/j.metop.2025.100400)
Supplement: Multimedia component 1 [file mmc1.docx]

**Supplementary Table 1:** Pathway-Specific Cognitive Trajectory Analysis and Metabolic Modulation.

| **Pathway Stage** | **Trajectory Characteristics** | **MMSE** | **ADAS** | **Metabolic Modulation** | **Implications** |
| --- | --- | --- | --- | --- | --- |
| **Cognitively Normal Baseline:** | | | | | |
| Participants/Visits | 3,312 participants | --- | --- | --- | Preclinical trajectory |
| Annual decline rate | Mean: -0.52 ± 1.68 | -0.52 pts/year | +0.91 pts/year | Baseline reference | Stable cognitive function |
| Trajectory variability | High individual differences | SD: 1.68 | SD: 2.6 | --- | Heterogeneous progression |
| **MCI Pathway Dynamics:** | | | | | |
| Participants/Visits | 379/1,707 | --- | --- | --- | Sequential decline pattern |
| Trajectory acceleration | 2.7× faster than CN | -1.38 pts/year | +3.66 pts/year | --- | Moderate progression |
| Time effect | β = -0.613 (0.102) | p < 0.001 | --- | --- | Significant linear decline |
| FDG protection effect | β = 1.842 (0.178) | p < 0.001 | --- | --- | Metabolic protection |
| Time×FDG interaction | β = 0.652 (0.109) | p = 2.21e-9 | --- | Moderate modulation | Metabolic trajectory influence |
| **AD Pathway Dynamics:** | | | | | |
| Participants/Visits | 170/500 | --- | --- | --- | Rapid conversion pattern |
| Trajectory acceleration | 5.6× faster than CN | -2.9 pts/year | +6.26 pts/year | --- | Accelerated progression |
| Time effect | β = -1.949 (0.31) | p < 0.001 | --- | --- | Steep linear decline |
| FDG protection effect | β = 1.396 (0.296) | p < 0.001 | --- | --- | Strong metabolic protection |
| Time×FDG interaction | β = 1.513 (0.201) | p = 4.74e-14 | --- | Strong modulation | Powerful metabolic effect |
| **Cross-Pathway Comparisons:** | | | | | |
| MCI vs AD acceleration | 2.1× steeper in AD | --- | --- | --- | Pathway-dependent velocity |
| Metabolic modulation strength | AD > MCI pathway | --- | --- | 2.3× stronger in AD | Increasing FDG importance |
| Trajectory predictability | Decreases with severity | --- | --- | --- | Early intervention optimal |

***Notes:*** *Mixed-effects model results show pathway-specific coefficients. Time×FDG interaction quantifies metabolic modulation of cognitive trajectories. Stronger interactions indicate greater metabolic influence on decline rates. Trajectory acceleration calculated relative to cognitively normal baseline.* ***Abbreviations:*** *CN, cognitively normal; MCI, mild cognitive impairment; AD, Alzheimer's disease; MMSE, Mini-Mental State Examination; ADAS, Alzheimer's Disease Assessment Scale; FDG, fluorodeoxyglucose positron emission tomography; SE, standard error; pts, points.*
