# Supplementary material for: FDG-PET brain glucose hypometabolism predicts Alzheimer's disease progression pathways in cognitively normal adults: A longitudinal competing risks modeling
Source: Metabol Open. 2025 Sep 26;28:100400. doi: 10.1016/j.metop.2025.100400 (PMC12516547; doi:10.1016/j.metop.2025.100400)
Supplement: Multimedia component 2 [file mmc2.docx]

**Supplementary Table 2:** Bootstrap Validation and Population Health Impact Assessment.

| **Validation Metric** | **Pathway Analysis** | **Bootstrap Results** | **Population Impact** | **Significance** |
| --- | --- | --- | --- | --- |
| **Bootstrap Validation of Pathway Models:** | | | | |
| Direct AD pathway risk ratio | Point estimate: 3.79 | Bootstrap mean: 3.82 ± 0.45 | 95% CI: [2.94, 4.88] | Highly significant effect |
|  | 1000 bootstrap iterations | Bias-corrected estimate | --- | Statistical confidence |
| Sequential MCI pathway risk ratio | Point estimate: 1.43 | Bootstrap mean: 1.44 ± 0.12 | 95% CI: [1.21, 1.68] | Moderate significant effect |
|  | 1000 bootstrap iterations | Stable across resamples | --- | Consistent pathway effect |
| **Cross-Validation Performance:** | | | | |
| Pathway prediction accuracy | MMSE model RMSE: 3.387 | 95.4% PI coverage | MAE: 2.37 | Excellent calibration |
|  | ADAS model RMSE: 9.565 | 95.3% PI coverage | MAE: 7.225 | Good generalizability |
| Validation method | 5-fold grouped by subject | Subject-grouped design | Prevents data leakage | Unbiased estimates |
| **Population Health Impact (US 65+ Population):** | | | | |
| Direct AD pathway prevention | Baseline: 6.5M cases/year | Preventable: 5.6M cases/year | 86.5% relative reduction | Major public health impact |
| Population NNT | 7 per 100 person-years | Metabolic intervention benefit | Cost-effective threshold | Healthcare resource planning |
| MCI pathway prevention | Baseline: 10.4M cases/year | Preventable: 5.6M cases/year | 54.4% relative reduction | Significant impact |
| Population NNT | 7 per 100 person-years | Improved quality of life | Family burden reduction | Societal benefit |
| **Pathway-Specific Validation:** | | | | |
| Bootstrap stability index | Direct AD: 0.94 | MCI: 0.89 | Stable: 0.96 | High reproducibility |
| Cross-validation consistency | AUC variance: 0.003 | Calibration stable | Pathway effects robust | Clinical reliability |
| Threshold significance | ±0.1 FDG z-score | Classification stable | Decision boundaries clear | Implementation confidence |

***Notes:*** *Bootstrap validation used bias-corrected accelerated (BCa) confidence intervals. Population impact calculated for US adults ≥65 years (54.1 million, 70% cognitively normal). Economic estimates based on lifetime care costs and intervention costs. PI: prediction interval; NNT: number needed to treat; MAE: mean absolute error; RMSE: root mean square error.* ***Abbreviations:*** *AD, Alzheimer's disease; MCI, mild cognitive impairment; CI, confidence interval; FDG, fluorodeoxyglucose positron emission tomography; MMSE, Mini-Mental State Examination; ADAS, Alzheimer's Disease Assessment Scale; AUC, area under curve; PY, person-years.*
