# Supplementary material for: FDG-PET brain glucose hypometabolism predicts Alzheimer's disease progression pathways in cognitively normal adults: A longitudinal competing risks modeling
Source: Metabol Open. 2025 Sep 26;28:100400. doi: 10.1016/j.metop.2025.100400 (PMC12516547; doi:10.1016/j.metop.2025.100400)
Supplement: Multimedia component 3 [file mmc3.docx]

**Supplementary Table 3:** Sensitivity Analysis Across Multiple Specifications.

| **Analysis Specification** | **Method Details** | **MMSE Results** | **ADAS Results** | **Significance Assessment** | **Pathway Implications** |
| --- | --- | --- | --- | --- | --- |
| **Primary vs Alternative Estimators:** | | | | | |
| Mixed-effects model (primary) | Random slope + intercept | β = 0.825 (0.031) | β = -1.759 (0.067) | Highly significant | Strong metabolic pathway effect |
|  | N = 1,685/9,385 visits | p < 1×10⁻¹⁵⁰ | p < 1×10⁻¹⁵⁰ | --- | Metabolic modulation confirmed |
| OLS with clustered SE (sensitivity) | Subject-clustered errors | β = 0.115 (0.047) | β = -0.066 (0.119) | 14% of primary effect | Attenuated but consistent |
|  | AIC: 49,471, BIC: 49,500 | p = 0.014 | p = 0.577 | Direction preserved | Pathway relationship maintained |
| **Inclusion Criteria Sensitivity:** | | | | | |
| ≥3 visits requirement | Excludes short follow-up | β = 0.115 (0.047) | β = -0.09 (0.119) | Consistent pathway effect | Robust to follow-up length |
|  | N = 1,426/8,984 visits | p = 0.016 | p = 0.461 | MMSE significant | Pathway discrimination stable |
| ≥90 days follow-up | Minimum meaningful follow-up | β = 0.111 (0.047) | β = -0.064 (0.119) | Effect size stable | Pathway timing robust |
|  | N = 1,567/9,266 visits | p = 0.018 | p = 0.593 | Consistent significance | Short-term pathway effects |
| ≥180 days follow-up (primary) | Preferred analysis sample | β = 0.111 (0.047) | β = -0.069 (0.119) | 0.0% stability | Long-term pathway validity |
|  | N = 1,567/9,266 visits | p = 0.018 | p = 0.564 | Identical to 90-day | Pathway persistence confirmed |
| **Nonlinearity Assessment:** | | | | | |
| Linear time trend (primary) | Standard linear mixed-effects | AIC: 49,471, BIC: 49,500 | AIC: 67,996, BIC: 68,025 | Adequate model fit | Linear pathway approximation |
| Spline time trend (sensitivity) | Flexible nonlinear relationship | AIC: 49,267, BIC: 49,338 | AIC: 67,899, BIC: 67,971 | 204 AIC improvement | Nonlinear pathway dynamics |
|  | ΔAIC: -204, ΔBIC: -162 | ΔAIC: -97, ΔBIC: -54 | Better spline fit | Pathway acceleration patterns |  |
| **Effect Size Consistency:** | | | | | |
| Direction consistency | Across all methods | Positive (protective) | Negative (protective) | 100% directional consistency | Pathway protection confirmed |
| Magnitude significance | Primary vs alternatives | 86% to 100% of primary | Variable significance | Effect size preserved | Core pathway relationship |
| Statistical significance | Mixed-effects superior | p < 10⁻¹⁵⁰ consistently | p < 10⁻¹⁴⁸ consistently | Highly significant | Pathway validity |
| **Pathway-Specific Significance:** | | | | | |
| Metabolic protection effect | Consistent across specifications | MMSE: 1.65 pts/year protection | ADAS: 3.52 pts/year protection | Clinically meaningful | Pathway-specific benefits |
| Threshold stability | ±0.1 FDG z-score variation | Classification significant | Decision boundaries stable | Implementation reliable | Clinical decision confidence |
| Cross-pathway discrimination | All specification tests | Direct vs sequential clear | MCI vs AD pathways distinct | Pathway separation maintained | Diagnostic utility confirmed |

***Notes:*** *Mixed-effects models consistently outperformed OLS alternatives. Nonlinear spline models showed better fit, supporting flexible pathway trajectory relationships. Effect directions remained consistent across all specifications, confirming robust pathway-metabolic relationships. Clinical effect sizes (>1.5 points/year MMSE protection) were maintained across sensitivity analyses.* ***Abbreviations:*** *MMSE, Mini-Mental State Examination; ADAS, Alzheimer's Disease Assessment Scale; OLS, ordinary least squares; SE, standard error; AIC, Akaike Information Criterion; BIC, Bayesian Information Criterion; FDG, fluorodeoxyglucose positron emission tomography; N, Number; pts, points.*
