# Supplementary material for: FDG-PET brain glucose hypometabolism predicts Alzheimer's disease progression pathways in cognitively normal adults: A longitudinal competing risks modeling
Source: Metabol Open. 2025 Sep 26;28:100400. doi: 10.1016/j.metop.2025.100400 (PMC12516547; doi:10.1016/j.metop.2025.100400)
Supplement: Multimedia component 4 [file mmc4.docx]

**Supplementary Table 4:** Monte Carlo Simulation and Cross-Validation Performance.

| **Validation Component** | **Performance Metrics** | **Monte Carlo Results** | **Stability Assessment** | **Reliability** |
| --- | --- | --- | --- | --- |
| **Cross-Validation Performance:** | | | | |
| MMSE pathway model | RMSE: 3.387 (11.3% of scale) | 95.4% PI coverage | Random effects σ²: 4.439 | Excellent calibration |
|  | MAE: 2.37 | 5-fold grouped by subject | N: 1,685/9,385 | Clinical precision adequate |
| ADAS pathway model | RMSE: 9.565 (13.7% of scale) | 95.3% PI coverage | Random effects σ²: 58.388 | Good calibration |
|  | MAE: 7.225 | 5-fold grouped by subject | N: 1,678/9,250 | Pathway discrimination reliable |
| **Monte Carlo Pathway Stability:** | | | | |
| Direct AD pathway risk | Mean RR: 3.82 | MC 95% CI: [3.34, 4.31] | Stability: 0.94 | Highly stable pathway effect |
|  | MC variance: 0.24 | 10,000 iterations | --- | Significant across simulations |
| Sequential MCI pathway risk | Mean RR: 1.44 | MC 95% CI: [1.28, 1.61] | Stability: 0.89 | Moderate stability |
|  | MC variance: 0.08 | 10,000 iterations | --- | Consistent pathway signal |
| Cognitive stability pathway | Mean protection: 0.57 | MC 95% CI: [0.47, 0.67] | Stability: 0.96 | Highest stability |
|  | MC variance: 0.05 | 10,000 iterations | --- | Preservation pathway significant |
| **Pathway Discrimination Performance:** | | | | |
| Direct AD conversion prediction | Mean AUC: 0.996 | 12m: 0.999, 24m: 0.994, 36m: 0.994 | Excellent discrimination | Clinical decision support |
|  | Mean Brier: 0.007 | Calibration consistent | --- | High-confidence predictions |
| MCI conversion prediction | Mean AUC: 0.670 | 12m: 0.686, 24m: 0.643, 36m: 0.68 | Acceptable discrimination | Screening utility |
|  | Mean Brier: 0.241 | Stable calibration | --- | Reliable probability estimates |
| **Threshold Significance Assessment:** | | | | |
| Optimal pathway cutpoint | FDG z-score: -0.11 | Sensitivity range: [-0.21, -0.01] | Significance: 0.87 | Sequential vs direct separation |
| Classification stability | ±0.1 SD threshold variation | 92% stable classifications | --- | Clinical implementation reliable |
| Decision boundary confidence | Monte Carlo validated | 1000 bootstrap replications | Threshold confidence: 92% | Diagnostic utility confirmed |
| **Temporal Validation Performance:** | | | | |
| Time-series cross-validation | Train: 2005-2015, Test: 2016-2020 | AUC degradation: <5% | Temporal stability: 0.89 | Generalizable across time |
| Pathway prediction consistency | 12m vs 36m horizon | Correlation: r = 0.94 | --- | Stable long-term predictions |
| Metabolic effect persistence | Across validation folds | CV coefficient: 0.03 | Low inter-fold variability | Significant metabolic-pathway link |

***Notes:*** *Cross-validation used subject-grouped 5-fold design to prevent optimistic bias. Monte Carlo simulations used 10,000 iterations with parametric bootstrapping. Pathway stability indices >0.85 indicate robust effects suitable for clinical implementation. Temporal validation confirms model generalizability across different time periods.* ***Abbreviations:*** *MMSE, Mini-Mental State Examination; ADAS, Alzheimer's Disease Assessment Scale; CV, cross-validation; PI, prediction interval; MC, Monte Carlo; RR, risk ratio; CI, confidence interval; AUC, area under curve; FDG, fluorodeoxyglucose positron emission tomography; AD, Alzheimer's disease; MCI, mild cognitive impairment; RMSE, root mean square error; MAE, mean absolute error; N, Number.*
