# Supplementary material for: FDG-PET brain glucose hypometabolism predicts Alzheimer's disease progression pathways in cognitively normal adults: A longitudinal competing risks modeling
Source: Metabol Open. 2025 Sep 26;28:100400. doi: 10.1016/j.metop.2025.100400 (PMC12516547; doi:10.1016/j.metop.2025.100400)
Supplement: Multimedia component 5 [file mmc5.docx]

**Supplementary Table 5:** Metabolic Threshold Discovery Analysis.

| **Threshold Analysis** | **FDG Z-Score Range** | **Sensitivity/Specificity** | **Performance** | **Pathway Discrimination** |
| --- | --- | --- | --- | --- |
| **Cognitive Stability vs Decline:** | | | | |
| Optimal threshold | +0.50 SD | Sensitivity: 73%, Specificity: 68% | Youden index: 0.41 | Cognitive preservation predictor |
| High stability (>+0.5) | FDG z-score >+0.5 | 24m stability: 55.4% | AD risk: 6.3% | Resilient metabolic phenotype |
| Low stability (<+0.5) | FDG z-score <+0.5 | Increased decline risk | Combined AD+MCI: >60% | Vulnerable metabolic profile |
| **Sequential vs Direct Pathway:** | | | | |
| Pathway switch threshold | -0.11 SD | Sensitivity: 62%, Specificity: 71% | Youden index: 0.33 | MCI vs direct AD separator |
| Sequential pathway favored | FDG z-score >-0.11 | Gradual decline expected | MCI-first pathway: 65% | Traditional progression |
| Direct pathway favored | FDG z-score <-0.11 | Rapid conversion risk | Direct AD pathway: 75% | Bypass MCI stage |
| **High-Risk Direct AD Conversion:** | | | | |
| High-risk threshold | -0.75 SD | Sensitivity: 78%, Specificity: 82% | Youden index: 0.60 | Direct conversion predictor |
| Very high risk (<-0.75) | FDG z-score <-0.75 | 24m AD risk: >30% | Intensive monitoring | Metabolic failure pattern |
| Moderate risk (-0.75 to -0.11) | Intermediate hypometabolism | 24m AD risk: 15-25% | Enhanced surveillance | Mixed pathway risk |
| **Dose-Response Analysis:** | | | | |
| FDG z-score: +2.0 | Preserved metabolism | Direct AD: 1%, MCI: 30% | Stability: 70% | Optimal metabolic reserve |
| FDG z-score: +1.0 | Good metabolism | Direct AD: 3%, MCI: 40% | Stability: 50% | Protective metabolism |
| FDG z-score: 0.0 | Average metabolism | Direct AD: 8%, MCI: 50% | Stability: 30% | Baseline risk profile |
| FDG z-score: -1.0 | Hypometabolism | Direct AD: 18%, MCI: 60% | Stability: 10% | Elevated pathway risk |
| FDG z-score: -2.0 | Severe hypometabolism | Direct AD: 25%, MCI: 70% | Stability: 5% | Metabolic crisis pattern |
| **ROC Curve:** | | | | |
| *Pathway discrimination AUC:* | | | | |
| Stability vs any decline | AUC: 0.74 (0.71-0.77) | Optimal threshold: +0.50 | Good discrimination | Clinical utility confirmed |
| Sequential vs direct AD | AUC: 0.67 (0.63-0.71) | Optimal threshold: -0.11 | Acceptable discrimination | Pathway guidance |
| Direct AD vs all others | AUC: 0.83 (0.80-0.86) | Optimal threshold: -0.75 | Excellent discrimination | High-risk identification |
| **Likelihood Ratios:** | | | | |
| High FDG (>+0.5) | Positive LR: 2.3 | Negative LR: 0.4 | Post-test probability ↓ | Reassuring metabolic profile |
| Low FDG (<-0.5) | Positive LR: 3.8 | Negative LR: 0.3 | Post-test probability ↑ | Concerning metabolic profile |
| Very low FDG (<-1.0) | Positive LR: 5.2 | Negative LR: 0.2 | High conversion risk | Urgent intervention candidate |
| **Segmented Regression Results:** | | | | |
| Metabolic breakpoint 1 | FDG z-score: +0.3 | Stability maintenance | --- | Cognitive reserve threshold |
| Metabolic breakpoint 2 | FDG z-score: -0.2 | Pathway acceleration | --- | Vulnerability emergence |
| Metabolic cliff | FDG z-score: -0.8 | Rapid progression risk | --- | Critical metabolic failure |
| **Implementation Thresholds:** | | | | |
| Screening threshold | FDG z-score: +0.25 | High sensitivity (85%) | Catch most at-risk | Population screening |
| Decision threshold | FDG z-score: -0.11 | Balanced performance | Clinical decisions | Pathway-specific care |
| Treatment threshold | FDG z-score: -0.75 | High specificity (90%) | Minimize false positives | Intensive interventions |

***Abbreviations:*** *FDG, fluorodeoxyglucose positron emission tomography; AD, Alzheimer's disease; MCI, mild cognitive impairment; ROC, receiver operating characteristic; AUC, area under curve; CI, confidence interval; LR, likelihood ratio; SD, standard deviation.*
