# Supplementary material for: FDG-PET brain glucose hypometabolism predicts Alzheimer's disease progression pathways in cognitively normal adults: A longitudinal competing risks modeling
Source: Metabol Open. 2025 Sep 26;28:100400. doi: 10.1016/j.metop.2025.100400 (PMC12516547; doi:10.1016/j.metop.2025.100400)
Supplement: Multimedia component 6 [file mmc6.docx]

**Supplementary Table 6:** Model Comparison and Selection Criteria.

| **Model Approach** | **Model Fit Statistics** | **Pathway Performance** | **Selection Criteria** | **Utility** |
| --- | --- | --- | --- | --- |
| **Primary Mixed-Effects Models:** | | | | |
| Linear mixed-effects (MMSE) | AIC: 49,471.5, BIC: 49,500.1 | Time×FDG: β=0.825 (0.031) | Random effects σ²: 4.439 | Strong pathway modulation |
|  | Log-likelihood: -24729.75 | p < 1×10⁻¹⁵⁰ | N: 1,685/9,385 visits | Clinical decision support |
| Linear mixed-effects (ADAS) | AIC: 67,996.2, BIC: 68,024.7 | Time×FDG: β=-1.759 (0.067) | Random effects σ²: 58.388 | Complementary pathway view |
|  | Log-likelihood: -33992.1 | p < 1×10⁻¹⁴⁸ | N: 1,678/9,250 visits | Convergent validity |
| **Alternative Estimation Methods:** | | | | |
| OLS with clustered SE | AIC: 49,471.5, BIC: 49,500.1 | Time×FDG: β=0.115 (0.047) | 14% of mixed-effects | Attenuated pathway effect |
|  | Ignores within-subject correlation | Direction preserved | Lower statistical power | Sensitivity analysis only |
| **Nonlinearity Comparison:** | | | | |
| Linear time trend | AIC: 49,471.5, BIC: 49,500.1 | Constant trajectory slopes | Adequate baseline fit | Simple interpretation |
| Spline time trend | AIC: 49,266.6, BIC: 49,338.1 | Flexible trajectory patterns | ΔAIC: -204.9 (MMSE) | Pathway acceleration patterns |
|  | ΔBIC: -161.9 (MMSE) | Nonlinear relationships | ΔAIC: -96.7 (ADAS) | Better empirical fit |
| **Pathway-Specific Models:** | | | | |
| Multinomial pathway classification | McFadden R²: 0.28 | Pathway accuracy by type: | Concordance: 0.74 | Multi-pathway discrimination |
|  | 4 pathway outcomes | Direct AD: 0.83 | --- | Excellent direct AD classification |
|  | Categorical outcome modeling | Sequential MCI: 0.67 | --- | Moderate MCI classification |
|  |  | Cognitive stability: 0.76 | --- | Good stability prediction |
| Competing risks (Fine-Gray) | AIC: 4267.3, BIC: 4298.7 | Direct AD C-index: 0.81 | Brier score: 0.12 | Time-to-event modeling |
|  | Subdistribution hazards | Sequential MCI C-index: 0.69 | Brier score: 0.24 | Competing pathway events |
| **Model Selection Results:** | | | | |
| AIC weights | Mixed-effects: 68% | Spline model: 25% | Primary model strongly favored | Statistical evidence clear |
|  | Competing risks: 5% | Multinomial: 2% | Alternative models secondary | Pathway focus confirmed |
| **Diagnostic Criteria:** | | | | |
| *Residual Analysis:* | | | | |
| Normality (Shapiro-Wilk) | Mixed-effects: p = 0.08 | Spline: p = 0.12 | Assumption satisfied | Significant inference |
| Homoscedasticity (Breusch-Pagan) | Mixed-effects: p = 0.15 | OLS: p = 0.003 | Random effects needed | Heterogeneity modeled |
| Independence (Durbin-Watson) | Mixed-effects: DW = 1.89 | OLS: DW = 0.67 | Correlation addressed | Longitudinal structure |
| *Assumption Testing:* | | | | |
| Linearity (pathway relationships) | Spline model superior | Nonlinear improvements significant | Pathway acceleration confirmed | Flexible trajectory modeling |
| Missing data (MCAR test) | p = 0.23 (not rejected) | Missing completely at random | Unbiased inference | Complete case analysis valid |
| Outlier detection (Cook's D) | 0.3% influential observations | Significant to outliers | Sensitivity confirmed | Clinical reliability |
| *Cross-Model Validation:* | | | | |
| Pathway prediction consistency | Mixed-effects ↔ Multinomial: r = 0.87 | Direct AD agreement: 91% | High inter-model agreement | Convergent validity |
| Effect size stability | Across estimation methods | Direction: 100% consistent | Magnitude: 85-100% preserved | Significant pathway relationship |
| Clinical decision concordance | Threshold-based classifications | Agreement: 89% | Kappa: 0.83 | Implementation reliable |

***Abbreviations:*** *AIC, Akaike Information Criterion; BIC, Bayesian Information Criterion; OLS, ordinary least squares; SE, standard error; MMSE, Mini-Mental State Examination; ADAS, Alzheimer's Disease Assessment Scale; FDG, fluorodeoxyglucose positron emission tomography; AD, Alzheimer's disease; MCI, mild cognitive impairment; MCAR, missing completely at random; N, Number; DW, Durbin-Watson statistic.*
